# Supplementary material for: We Are on the Verge of Breakthrough Cures for Type 1 Diabetes, but Who Are the 2 Million Americans Who Have It?
Source: J Health Econ Outcomes Res. 2024 Nov 12;11(2):145–53. doi: 10.36469/001c.124604 (PMC11566618; doi:10.36469/001c.124604)
Supplement: Online Supplementary Material [file jheor_2024_11_2_124604_251149.pdf]

APPENDIX

Exhibit A1. Population Sizes of Each Market in Data and in the Nation

Market	Population Size (in Data)	Population Size in Millions	Source
Commercial	10,769,405	11	Milliman Consolidated Health Cost Sources Database
Medicaid	4,040,095	4	Milliman Consolidated Health Cost Sources Database
Medicare Fee-for-Service	20,607,550	21	CMS 100% Research Identifiable Medicare Data
Medicare Advantage	20,776,377	21	CMS 100% Research Identifiable Medicare Encounter Data

Market	Population Size (in Literature)	Population Size in Millions	Source
Commercial	172,700,000	173	CDC National Center for Health Statistics
Medicaid	64,896,849	65	CMS Medicaid and CHIP Scorecard
Medicare Fee-for-Service	38,577,012	39	CMS Chronic Condition Warehouse
Medicare Advantage	22,200,000	22	Kaiser Family Foundation
Veterans Affairs	4,236,144	4	US Department of Veterans Affairs
Uninsured	32,800,000	33	CDC National Center for Health Statistics
Other Medicare Health Plan Enrollment	3,622,988	4	CMS Chronic Condition Warehouse

Exhibit A2. HCPCS Codes for Insulin, Insulin Pumps, and Insulin-Related DME

Code Type	Code	Description	Insulin Type	Include/Exclude from T1DM
HCPCS	A4225	Supplies for external insulin infusion pump, syringe type cartridge, sterile, each	Not insulin	Include - Insulin Pump-Related Supplies
HCPCS	A4226	Supplies for maintenance of insulin infusion pump with dosage rate adjustment using therapeutic contir	Not insulin	Include - Insulin Pump-Related Supplies
HCPCS	A4230	Infusion set for external insulin pump, non needle cannula type	Not insulin	Include - Insulin Pump-Related Supplies
HCPCS	A4231	Infusion set for external insulin pump, needle type	Not insulin	Include - Insulin Pump-Related Supplies
HCPCS	A4232	Syringe with needle for external insulin pump, sterile, 3 cc	Not insulin	Include - Insulin Pump-Related Supplies
HCPCS	A9274	External ambulatory insulin delivery system, disposable, each, includes all supplies and accessories	Short	Include - Disposble Insulin Pump (contains short acting insulin)
HCPCS	E0784	External ambulatory infusion pump, insulin	Not insulin	Include - Insulin Pump
HCPCS	E0787	External ambulatory infusion pump, insulin, dosage rate adjustment using therapeutic continuous gluco	Not insulin	Include - Insulin Pump
HCPCS	J1815	Injection, insulin, per 5 units	Short	Include - Short acting insulin for T1DM ID. May be used in an acute episode for patients without diabetes.
HCPCS	J1817	Insulin for administration through dme (i.e., insulin pump) per 50 units	Short	Include - Short acting insulin for T1DM ID
HCPCS	K0601	Replacement battery for external infusion pump owned by patient, silver oxide, 1.5 volt, each	Not insulin	Include - Insulin Pump-Related Supplies
HCPCS	K0602	Replacement battery for external infusion pump owned by patient, silver oxide, 3 volt, each	Not insulin	Include - Insulin Pump-Related Supplies
HCPCS	K0603	Replacement battery for external infusion pump owned by patient, alkaline, 1.5 volt, each	Not insulin	Include - Insulin Pump-Related Supplies
HCPCS	K0604	Replacement battery for external infusion pump owned by patient, lithium, 3.6 volt, each	Not insulin	Include - Insulin Pump-Related Supplies
HCPCS	K0605	Replacement battery for external infusion pump owned by patient, lithium, 4.5 volt, each	Not insulin	Include - Insulin Pump-Related Supplies
HCPCS	S1034	Artificial pancreas device system (e.g., low glucose suspend (lgs) feature) including continuous glucose monitor, blood glucose device, insulin pump and computer algorithm that communicates with all of the devices	Not insulin	Include - Insulin Pump
HCPCS	S1035	Sensor; invasive (e.g., subcutaneous), disposable, for use with artificial pancreas device system	Not insulin	Include - Insulin Pump-Related Supplies
HCPCS	S1036	Transmitter; External, For Use With Artificial Pancreas Device System	Not insulin	Include - Insulin Pump-Related Supplies
HCPCS	S1037	Receiver (Monitor); External, For Use With Artificial Pancreas Device System	Not insulin	Include - Insulin Pump-Related Supplies
HCPCS	S5550	Insulin, rapid onset, 5 units	Short	Include - Short acting insulin for T1DM ID. May be used in an acute episode for patients without diabetes.
HCPCS	S5551	Insulin, most rapid onset (lispro or aspart); 5 units	Short	Include - Short acting insulin for T1DM ID. May be used in an acute episode for patients without diabetes.
HCPCS	S5552	Insulin, intermediate acting (nph or lente); 5 units	Intermediate	Exclude from T1DM ID - Intermediate acting insulin. May be used in an acute episode for patients without diabetes.
HCPCS	S5553	Insulin, long acting; 5 units	Long	Exclude from T1DM ID - Long acting insulin. May be used in an acute episode for patients without diabetes.
HCPCS	S5560	Insulin delivery device, reusable pen; 1.5 ml size	Not insulin	Include - Insulin-Related Supplies
HCPCS	S5561	Insulin delivery device, reusable pen; 3 ml size	Not insulin	Include - Insulin-Related Supplies
HCPCS	S5565	Insulin cartridge for use in insulin delivery device other than pump; 150 units	Short	Include - Short acting insulin for T1DM ID
HCPCS	S5566	Insulin cartridge for use in insulin delivery device other than pump; 300 units	Short	Include - Short acting insulin for T1DM ID
HCPCS	S5570	Insulin delivery device, disposable pen (including insulin); 1.5 ml size	Short	Include - Short acting insulin for T1DM ID
HCPCS	S5571	Insulin delivery device, disposable pen (including insulin); 3 ml size	Short	Include - Short acting insulin for T1DM ID
HCPCS	S8490	Insulin syringes (100 syringes, any size)	Not insulin	Include - Insulin-Related Supplies

Exhibit A3. NDC Codes for Insulin, Insulin Pumps, and Insulin-Related DME

NDCs were generated for each of the description categories and insulin types listed below. The full list of NDCs is available from the authors.

Code Type	Description	Insulin Type	Include/Exclude from T1DM
NDC	Insulin Aspart Inj 100 Unit/ML	Short	Include - Short-acting insulin for T1DM ID
NDC	Insulin Aspart Soln Pen-injector 100 Unit/ML	Short	Include - Short-acting insulin for T1DM ID
NDC	Insulin Aspart Soln Cartridge 100 Unit/ML	Short	Include - Short-acting insulin for T1DM ID
NDC	Insulin Glargine Inj 100 Unit/ML	Long	Exclude from T1DM ID - Long acting insulin
NDC	Insulin Glargine Soln Pen-Injector 100 Unit/ML	Long	Exclude from T1DM ID - Long acting insulin
NDC	Insulin Glargine Soln Pen-Injector 300 Unit/ML	Long	Exclude from T1DM ID - Long acting insulin
NDC	Insulin Glulisine Inj 100 Unit/ML	Short	Include - Short-acting insulin for T1DM ID
NDC	Insulin Glulisine Soln Pen-Injector Inj 100 Unit/ML	Short	Include - Short-acting insulin for T1DM ID
NDC	Insulin Lispro Inj 100 Unit/ML	Short	Include - Short-acting insulin for T1DM ID
NDC	Insulin Lispro Soln Pen-injector 100 Unit/ML	Short	Include - Short-acting insulin for T1DM ID
NDC	Insulin Lispro Soln Pen-injector 200 Unit/ML	Short	Include - Short-acting insulin for T1DM ID
NDC	Insulin Lispro Soln Cartridge 100 Unit/ML	Short	Include - Short-acting insulin for T1DM ID
NDC	Insulin Detemir Inj 100 Unit/ML	Long	Exclude from T1DM ID - Long acting insulin
NDC	Insulin Detemir Soln Pen-injector 100 Unit/ML	Long	Exclude from T1DM ID - Long acting insulin
NDC	Insulin Degludec Inj 100 Unit/ML	Long	Exclude from T1DM ID - Long acting insulin
NDC	Insulin Degludec Soln Pen-Injector 100 Unit/ML	Long	Exclude from T1DM ID - Long acting insulin
NDC	Insulin Degludec Soln Pen-Injector 200 Unit/ML	Long	Exclude from T1DM ID - Long acting insulin
NDC	Insulin Regular (Human) Inj 100 Unit/ML	Short	Include - Short-acting insulin for T1DM ID
NDC	Insulin Regular (Human) Inj 500 Unit/ML	Short	Include - Short-acting insulin for T1DM ID
NDC	Insulin Regular (Human) Inhalation Powder 4 Unit/Cartridge	Short	Include - Short-acting insulin for T1DM ID
NDC	Insulin Regular (Human) Inhalation Powder 8 Unit/Cartridge	Short	Include - Short-acting insulin for T1DM ID
NDC	Insulin Regular (Human) Inhalation Powder 12 Unit/Cartridge	Short	Include - Short-acting insulin for T1DM ID
NDC	Insulin Regular (Human) Inhal Powd 4 (30) & 8 (60) Unit/Cart	Short	Include - Short-acting insulin for T1DM ID
NDC	Insulin Regular (Human) Inhal Powd 4 (60) & 8 (30) Unit/Cart	Short	Include - Short-acting insulin for T1DM ID
NDC	Insulin Regular (Human) Inhal Powd 4 (90) & 8 (90) Unit/Cart	Short	Include - Short-acting insulin for T1DM ID
NDC	Insulin Regular (Human) Inh Powd 8 (60) & 12 (30) Unit/Cart	Short	Include - Short-acting insulin for T1DM ID
NDC	Insulin Regular (Human) Inh Powd 8 (90) & 12 (90) Unit/Cart	Short	Include - Short-acting insulin for T1DM ID
NDC	Insulin Regular (Human) Inh Powd 4 & 8 & 12 Unit/Cart (60)	Short	Include - Short-acting insulin for T1DM ID
NDC	Insulin Regular (Human) Soln Pen-Injector 500 Unit/ML	Short	Include - Short-acting insulin for T1DM ID
NDC	Insulin Isophane (Human) Inj 100 Unit/ML	Short	Include - Short-acting insulin for T1DM ID
NDC	Insulin NPH (Human) (Isophane) Inj 100 Unit/ML	Intermediate	Include - Intermediate-acting insulin for T1DM ID
NDC	Insulin NPH (Human) (Isophane) Susp Pen-injector 100 Unit/ML	Intermediate	Include - Intermediate-acting insulin for T1DM ID
NDC	Insulin Aspart Prot & Aspart (Human) Inj 100 Unit/ML (70-30)	Short, Intermediate	Include - Short-acting insulin for T1DM ID
NDC	Insulin Aspart Prot & Aspart Sus Pen-inj 100 Unit/ML (70-30)	Short, Intermediate	Include - Short-acting insulin for T1DM ID
NDC	Insulin Lispro Prot & Lispro Inj 100 Unit/ML (75-25)	Short, Intermediate	Include - Short-acting insulin for T1DM ID
NDC	Insulin Lispro Protamine & Lispro Inj 100 Unit/ML (50-50)	Short, Intermediate	Include - Short-acting insulin for T1DM ID
NDC	Insulin Lispro Prot & Lispro Sus Pen-inj 100 Unit/ML (75-25)	Short, Intermediate	Include - Short-acting insulin for T1DM ID
NDC	Insulin Lispro Prot & Lispro Sus Pen-inj 100 Unit/ML (50-50)	Short, Intermediate	Include - Short-acting insulin for T1DM ID
NDC	Insulin Isophane & Regular (Human) Inj 100 Unit/ML (70-30)	Short, Intermediate	Include - Short-acting insulin for T1DM ID
NDC	Insulin NPH Isophane & Regular Human Inj 100 Unit/ML (70-30)	Short, Intermediate	Include - Short-acting insulin for T1DM ID
NDC	Insulin NPH & Regular Susp Pen-Inj 100 Unit/ML (70-30)	Short, Intermediate	Include - Short-acting insulin for T1DM ID
NDC	Insulin Degludec-Liraglutide Sol Pen-Inj 100-3.6 Unit-MG/ML	Long, GLP1	Exclude from T1DM ID - Long acting insulin
NDC	Insulin Glargine-Lixisenatide Sol Pen-Inj 100-33 Unit-MCG/ML	Long, GLP1	Exclude from T1DM ID - Long acting insulin
NDC	Insulin Syringe (Disp) U-100 0.3 ML	Not insulin	Include - Insulin-Related Supplies
NDC	Insulin Syringe (Disp) U-100 1/2 ML	Not insulin	Include - Insulin-Related Supplies
NDC	Insulin Syringe (Disp) U-100 1 ML	Not insulin	Include - Insulin-Related Supplies
NDC	Insulin Syringe/Needle U-40 1 ML 25 x 5/8"	Not insulin	Include - Insulin-Related Supplies
NDC	Insulin Syringe/Needle U-100 0.3 ML 29 G	Not insulin	Include - Insulin-Related Supplies
NDC	Insulin Syringe/Needle U-100 0.3 ML 30 G	Not insulin	Include - Insulin-Related Supplies
NDC	Insulin Syringe/Needle U-100 0.3 ML 28 x 1/2"	Not insulin	Include - Insulin-Related Supplies
NDC	Insulin Syringe/Needle U-100 0.3 ML 29 x 1/2"	Not insulin	Include - Insulin-Related Supplies
NDC	Insulin Syringe/Needle U-100 0.3 ML 30 x 3/8"	Not insulin	Include - Insulin-Related Supplies
NDC	Insulin Syringe/Needle U-100 0.3 ML 30 x 5/16"	Not insulin	Include - Insulin-Related Supplies
NDC	Insulin Syringe/Needle U-100 0.3 ML 30 x 1/2"	Not insulin	Include - Insulin-Related Supplies
NDC	Insulin Syringe/Needle U-100 1/2 ML 27 x 1/2"	Not insulin	Include - Insulin-Related Supplies
NDC	Insulin Syringe/Needle U-100 1/2 ML 29 G	Not insulin	Include - Insulin-Related Supplies
NDC	Insulin Syringe/Needle U-100 1/2 ML 30 G	Not insulin	Include - Insulin-Related Supplies
NDC	Insulin Syringe/Needle U-100 1/2 ML 30 x 3/8"	Not insulin	Include - Insulin-Related Supplies
NDC	Insulin Syringe/Needle U-100 1/2 ML 31 x 5/16"	Not insulin	Include - Insulin-Related Supplies
NDC	Insulin Syringe/Needle U-100 1/2 ML 28 x 5/16"	Not insulin	Include - Insulin-Related Supplies
NDC	Insulin Syringe/Needle U-100 1/2 ML 28 x 1/2"	Not insulin	Include - Insulin-Related Supplies
NDC	Insulin Syringe/Needle U-100 1/2 ML 29 x 5/16"	Not insulin	Include - Insulin-Related Supplies
NDC	Insulin Syringe/Needle U-100 1/2 ML 29 x 1/2"	Not insulin	Include - Insulin-Related Supplies
NDC	Insulin Syringe/Needle U-100 1/2 ML 30 x 5/16"	Not insulin	Include - Insulin-Related Supplies
NDC	Insulin Syringe/Needle U-100 1/2 ML 30 x 1/2"	Not insulin	Include - Insulin-Related Supplies
NDC	Insulin Syringe/Needle U-100 1 ML 25 x 5/8"	Not insulin	Include - Insulin-Related Supplies
NDC	Insulin Syringe/Needle U-100 1/2 ML 31 x 3/8"	Not insulin	Include - Insulin-Related Supplies
NDC	Insulin Syringe/Needle U-100 1 ML 31 x 3/8"	Not insulin	Include - Insulin-Related Supplies
NDC	Insulin Syringe/Needle U-100 0.3 ML 31 x 15/64"	Not insulin	Include - Insulin-Related Supplies
NDC	Insulin Syringe/Needle U-100 0.3 ML 31 x 1/4" (6 MM)	Not insulin	Include - Insulin-Related Supplies
NDC	Insulin Syringe/Needle U-100 1 ML 25 x 1"	Not insulin	Include - Insulin-Related Supplies
NDC	Insulin Syringe/Needle U-100 0.5 ML 31 x 1/4" (6 MM)	Not insulin	Include - Insulin-Related Supplies
NDC	Insulin Syringe/Needle U-100 1 ML 31 x 1/4" (6 MM)	Not insulin	Include - Insulin-Related Supplies
NDC	Insulin Syringe/Needle U-100 1 ML 30 x 3/16" (5 MM)	Not insulin	Include - Insulin-Related Supplies
NDC	Insulin Syringe/Needle U-100 1 ML 26 x 1/2"	Not insulin	Include - Insulin-Related Supplies
NDC	Insulin Syringe/Needle U-100 1 ML 27 x 1/2"	Not insulin	Include - Insulin-Related Supplies
NDC	Insulin Syringe/Needle U-100 0.5 ML 30 x 3/16" (5 MM)	Not insulin	Include - Insulin-Related Supplies
NDC	Insulin Syringe/Needle U-100 0.3 ML 30 x 15/64"	Not insulin	Include - Insulin-Related Supplies
NDC	Insulin Syringe/Needle U-100 1 ML 27 x 5/8"	Not insulin	Include - Insulin-Related Supplies
NDC	Insulin Syringe/Needle U-100 0.5 ML 30 x 15/64"	Not insulin	Include - Insulin-Related Supplies
NDC	Insulin Syringe/Needle U-100 1 ML 30 x 15/64"	Not insulin	Include - Insulin-Related Supplies

Exhibit A3. NDC Codes for Insulin, Insulin Pumps, and Insulin-Related DME

NDCs were generated for each of the description categories and insulin types listed below. The full list of NDCs is available from the authors.

Code Type	Description	Insulin Type	Include/Exclude from T1DM
NDC	Insulin Aspart Inj 100 Unit/ML	Short	Include - Short-acting insulin for T1DM ID
NDC	Insulin Syringe/Needle U-100 1 ML 28 x 5/16"	Not insulin	Include - Insulin-Related Supplies
NDC	Insulin Syringe/Needle U-100 1 ML 28 x 1/2"	Not insulin	Include - Insulin-Related Supplies
NDC	Insulin Syringe/Needle U-100 1 ML 29 G	Not insulin	Include - Insulin-Related Supplies
NDC	Insulin Syringe/Needle U-100 1 ML 29 x 1/2"	Not insulin	Include - Insulin-Related Supplies
NDC	Insulin Syringe/Needle U-100 1 ML 29 x 5/16"	Not insulin	Include - Insulin-Related Supplies
NDC	Insulin Syringe/Needle U-100 1 ML 30 G	Not insulin	Include - Insulin-Related Supplies
NDC	Insulin Syringe/Needle U-100 1 ML 30 x 5/16"	Not insulin	Include - Insulin-Related Supplies
NDC	Insulin Syringe/Needle U-100 1 ML 30 x 1/2"	Not insulin	Include - Insulin-Related Supplies
NDC	Insulin Syringe/Needle U-100 1 ML 31 x 5/16"	Not insulin	Include - Insulin-Related Supplies
NDC	Insulin Syringe/Needle U-100 0.3 ML 31 x 5/16"	Not insulin	Include - Insulin-Related Supplies
NDC	Insulin Syringe/Needle U-100 0.3 ML 31 x 3/8"	Not insulin	Include - Insulin-Related Supplies
NDC	Insulin Syringe/Needle U-100 2 ML 27.5 x 5/8"	Not insulin	Include - Insulin-Related Supplies
NDC	Insulin Syringe/Needle U-100 1/2 ML 31 x 15/64"	Not insulin	Include - Insulin-Related Supplies
NDC	Insulin Syringe/Needle U-100 2 ML 29 x 1/2"	Not insulin	Include - Insulin-Related Supplies
NDC	Insulin Syringe/Needle U-100 1 ML 30 x 3/8"	Not insulin	Include - Insulin-Related Supplies
NDC	Insulin Syringe/Needle U-100 0.3 ML 29 x 1"	Not insulin	Include - Insulin-Related Supplies
NDC	Insulin Syringe/Needle U-100 1 ML 31 x 15/64"	Not insulin	Include - Insulin-Related Supplies
NDC	Insulin Syringe/Needle U-500 0.5 ML 31G x 6MM (15/64")	Not insulin	Include - Insulin-Related Supplies
NDC	Injection Device - Misc	Short	Include - Short-acting insulin for T1DM ID
NDC	Injection Device for Insulin	Not insulin	Include - Insulin-Related Supplies
NDC	Injection Device for Insulin	Short	Include - Short-acting insulin for T1DM ID
NDC	Insulin Pen Needle 29 G X 5 MM (3/16")	Not insulin	Include - Insulin-Related Supplies
NDC	Insulin Pen Needle 29 G X 8 MM (5/16")	Not insulin	Include - Insulin-Related Supplies
NDC	Insulin Pen Needle 29 G X 10 MM	Not insulin	Include - Insulin-Related Supplies
NDC	Insulin Pen Needle 29 G X 12 MM (1/2")	Not insulin	Include - Insulin-Related Supplies
NDC	Insulin Pen Needle 29 G X 12.7 MM	Not insulin	Include - Insulin-Related Supplies
NDC	Insulin Pen Needle 29 G X 13 MM (1/2")	Not insulin	Include - Insulin-Related Supplies
NDC	Insulin Pen Needle 30 G X 5 MM (3/16")	Not insulin	Include - Insulin-Related Supplies
NDC	Insulin Pen Needle 30 G X 8 MM (1/3" or 5/16")	Not insulin	Include - Insulin-Related Supplies
NDC	Insulin Pen Needle 31 G X 4 MM (1/6")	Not insulin	Include - Insulin-Related Supplies
NDC	Insulin Pen Needle 31 G X 5 MM (3/16")	Not insulin	Include - Insulin-Related Supplies
NDC	Insulin Pen Needle 31 G X 6 MM (1/4")	Not insulin	Include - Insulin-Related Supplies
NDC	Insulin Pen Needle 31 G X 8 MM (1/3" or 5/16")	Not insulin	Include - Insulin-Related Supplies
NDC	Insulin Pen Needle 32 G X 4 MM (5/32")	Not insulin	Include - Insulin-Related Supplies
NDC	Insulin Pen Needle 32 G X 5 MM (1/5" or 3/16")	Not insulin	Include - Insulin-Related Supplies
NDC	Insulin Pen Needle 32 G X 6 MM (1/4")	Not insulin	Include - Insulin-Related Supplies
NDC	Insulin Pen Needle 32 G X 8 MM	Not insulin	Include - Insulin-Related Supplies
NDC	Insulin Pen Needle 33 G X 4 MM (5/32")	Not insulin	Include - Insulin-Related Supplies
NDC	Insulin Pen Needle 33 G X 5 MM (1/5" or 3/16")	Not insulin	Include - Insulin-Related Supplies
NDC	Insulin Pen Needle 33 G X 6 MM (1/4")	Not insulin	Include - Insulin-Related Supplies
NDC	Insulin Pen Needle 33 G X 8 MM (1/3" or 5/16")	Not insulin	Include - Insulin-Related Supplies
NDC	Injection Device Needle-Free for Insulin	Not insulin	Include - Insulin-Related Supplies
NDC	Injection Device Needle-Free for Insulin Supplies	Not insulin	Include - Insulin-Related Supplies
NDC	Injection Device Needle-Free for Insulin Kit	Not insulin	Include - Insulin-Related Supplies
NDC	Insulin Administration Supplies - Misc	Not insulin	Include - Insulin-Related Supplies
NDC	Insulin Administration Supplies - Kit	Not insulin	Include - Insulin-Related Supplies
NDC	Insulin Infusion Pump - Device	Not insulin	Include - Insulin Pump
NDC	Insulin Infusion Pump - Kit	Not insulin	Include - Insulin Pump
NDC	Insulin Infusion Pump - Accessories	Not insulin	Include - Insulin Pump-Related Supplies
NDC	Insulin Infusion Pump Supplies	Not insulin	Include - Insulin Pump-Related Supplies
NDC	Insulin Infusion Disposable Pump Supplies	Short	Include - Disposable Insulin Pump (contains short-acting insulin)
NDC	Insulin Infusion Disposable Pump Kit	Short	Include - Disposable Insulin Pump (contains short-acting insulin)
NDC	Insulin Aspart (with Niacinamide) Soln Cartridge 100 Unit/ML	Short	Include - Short-acting insulin for T1DM ID
NDC	Insulin Glargine Soln Pen-Injector 300 Unit/ML (1 Unit Dial)	Long	Exclude from T1DM ID - Long acting insulin
NDC	Insulin Glargine Soln Pen-Injector 300 Unit/ML (2 Unit Dial)	Long	Exclude from T1DM ID - Long acting insulin
NDC	Insulin Lispro Soln Pen-injector 100 Unit/ML (0.5 Unit Dial)	Short	Include - Short-acting insulin for T1DM ID
NDC	Insulin Lispro Soln Pen-injector 100 Unit/ML (1 Unit Dial)	Short	Include - Short-acting insulin for T1DM ID
NDC	Insulin Regular (Human) Soln Pen-Injector 100 Unit/ML	Short	Include - Short-acting insulin for T1DM ID
NDC	Insulin Regular (Human) in NaCl 0.9% IV Soln 100 Unit/100ML	Short	Include - Short-acting insulin for T1DM ID
NDC	*Injection Device - Kit***	Short	Include - Insulin patch (incuding short acting insulin so used for T1DM ID)
NDC	Injection Device for Insulin	Short	Include - Insulin patch (incuding short acting insulin so used for T1DM ID)
NDC	*Injection Device for Insulin - Accessories***	Short	Include - Insulin patch (incuding short acting insulin so used for T1DM ID)
NDC	Insulin Pen Needle 29 G X 5 MM (1/5" or 3/16")	Not insulin	Include - Insulin-Related Supplies
NDC	Insulin Pen Needle 29 G X 8 MM (1/3" or 5/16")	Not insulin	Include - Insulin-Related Supplies
NDC	Insulin Pen Needle 29 G X 12.7 MM (1/2")	Not insulin	Include - Insulin-Related Supplies
NDC	Insulin Pen Needle 30 G X 5 MM (1/5" or 3/16")	Not insulin	Include - Insulin-Related Supplies
NDC	Insulin Pen Needle 31 G X 5 MM (1/5" or 3/16")	Not insulin	Include - Insulin-Related Supplies
NDC	Insulin Pen Needle 31 G X 6 MM (1/4" or 15/64")	Not insulin	Include - Insulin-Related Supplies
NDC	Insulin Pen Needle 32 G X 4 MM (1/6" or 5/32")	Not insulin	Include - Insulin-Related Supplies
NDC	Insulin Pen Needle 32 G X 6 MM (1/4" or 15/64")	Not insulin	Include - Insulin-Related Supplies
NDC	Insulin Pen Needle 32 G X 8 MM (1/3" or 5/16")	Not insulin	Include - Insulin-Related Supplies
NDC	Insulin Pen Needle 33 G X 4 MM (1/6" or 5/32")	Not insulin	Include - Insulin-Related Supplies
NDC	Insulin Pen Needle 33 G X 6 MM (1/4" or 15/64")	Not insulin	Include - Insulin-Related Supplies
NDC	*Insulin Infusion Pump - Device***	Not insulin	Include - Insulin Pump
NDC	*Insulin Infusion Pump Supplies***	Not insulin	Include - Insulin Pump-Related Supplies
NDC	Insulin Aspart (with Niacinamide) Inj 100 Unit/ML	Short	Include - Short-acting insulin for T1DM ID
NDC	Insulin Aspart (with Niacinamide) Sol Pen-inj 100 Unit/ML	Short	Include - Short-acting insulin for T1DM ID
NDC	Insulin Lispro-aabc Inj 100 Unit/ML	Short	Include - Short-acting insulin for T1DM ID
NDC	Insulin Lispro-aabc Soln Pen-inj 100 Unit/ML (1 Unit Dial)	Short	Include - Short-acting insulin for T1DM ID
NDC	Insulin Lispro-aabc Soln Pen-injector 200 Unit/ML	Short	Include - Short-acting insulin for T1DM ID
NDC	Insulin Regular (Human) Inhal Powd 30 x 4 Unit & 60 x 8 Unit	Short	Include - Short-acting insulin for T1DM ID

Exhibit A3. NDC Codes for Insulin, Insulin Pumps, and Insulin-Related DME

NDCs were generated for each of the description categories and insulin types listed below. The full list of NDCs is available from the authors.

Code Type	Description	Insulin Type	Include/Exclude from T1DM
NDC	Insulin Aspart Inj 100 Unit/ML	Short	Include - Short-acting insulin for T1DM ID
NDC	Insulin Regular (Human) Inhal Powd 60 x 4 Unit & 30 x 8 Unit	Short	Include - Short-acting insulin for T1DM ID
NDC	Insulin Regular (Human) Inhal Powd 90 x 4 Unit & 90 x 8 Unit	Short	Include - Short-acting insulin for T1DM ID
NDC	Insulin Regular (Human) Inh Powd 60 x 8 Unit & 30 x 12 Unit	Short	Include - Short-acting insulin for T1DM ID
NDC	Insulin Regular (Human) Inh Powd 90 x 8 Unit & 90 x 12 Unit	Short	Include - Short-acting insulin for T1DM ID
NDC	Insulin Syringe/Needle U-100 0.5 ML 32 x 5/16"	Not insulin	Include - Insulin-Related Supplies
NDC	Insulin Syringe/Needle U-100 1 ML 32 x 5/16"	Not insulin	Include - Insulin-Related Supplies
NDC	*Injection Device for Insulin Kit***	Short	Include - Insulin patch (incuding short acting insulin so used for T1DM ID)
NDC	Insulin Pen Needle 31 G X 4 MM (1/6" or 5/32")	Not insulin	Include - Insulin-Related Supplies
NDC	Insulin Pen Needle 34 G X 3.5 MM (9/64")	Not insulin	Include - Insulin-Related Supplies
NDC	Insulin Infusion Pump Supplies - Infusion Set	Not insulin	Include - Insulin Pump-Related Supplies
NDC	Insulin Infusion Pump Supplies - Reservoir	Not insulin	Include - Insulin Pump-Related Supplies

Exhibit A4. ICD-10-CM Diagnosis Codes for Type 1 Diabetes

Code Type	Code	Description
ICD-10-CM	E1010	Type 1 diabetes mellitus with ketoacidosis without coma
ICD-10-CM	E1011	Type 1 diabetes mellitus with ketoacidosis with coma
ICD-10-CM	E1021	Type 1 diabetes mellitus with diabetic nephropathy
ICD-10-CM	E1022	Type 1 diabetes mellitus with diabetic chronic kidney disease
ICD-10-CM	E1029	Type 1 diabetes mellitus with other diabetic kidney complication
ICD-10-CM	E10311	Type 1 diabetes mellitus with unspecified diabetic retinopathy with macular edema
ICD-10-CM	E10319	Type 1 diabetes mellitus with unspecified diabetic retinopathy without macular edema
ICD-10-CM	E10321	Type 1 diabetes mellitus with mild nonproliferative diabetic retinopathy with macular edema
ICD-10-CM	E103211	Type 1 diabetes mellitus with mild nonproliferative diabetic retinopathy with macular edema, right eye
ICD-10-CM	E103212	Type 1 diabetes mellitus with mild nonproliferative diabetic retinopathy with macular edema, left eye
ICD-10-CM	E103213	Type 1 diabetes mellitus with mild nonproliferative diabetic retinopathy with macular edema, bilateral
ICD-10-CM	E103219	Type 1 diabetes mellitus with mild nonproliferative diabetic retinopathy with macular edema, unspecified eye
ICD-10-CM	E10329	Type 1 diabetes mellitus with mild nonproliferative diabetic retinopathy without macular edema
ICD-10-CM	E103291	Type 1 diabetes mellitus with mild nonproliferative diabetic retinopathy without macular edema, right eye
ICD-10-CM	E103292	Type 1 diabetes mellitus with mild nonproliferative diabetic retinopathy without macular edema, left eye
ICD-10-CM	E103293	Type 1 diabetes mellitus with mild nonproliferative diabetic retinopathy without macular edema, bilateral
ICD-10-CM	E103299	Type 1 diabetes mellitus with mild nonproliferative diabetic retinopathy without macular edema, unspecified eye
ICD-10-CM	E10331	Type 1 diabetes mellitus with moderate nonproliferative diabetic retinopathy with macular edema
ICD-10-CM	E103311	Type 1 diabetes mellitus with moderate nonproliferative diabetic retinopathy with macular edema, right eye
ICD-10-CM	E103312	Type 1 diabetes mellitus with moderate nonproliferative diabetic retinopathy with macular edema, left eye
ICD-10-CM	E103313	Type 1 diabetes mellitus with moderate nonproliferative diabetic retinopathy with macular edema, bilateral
ICD-10-CM	E103319	Type 1 diabetes mellitus with moderate nonproliferative diabetic retinopathy with macular edema, unspecified eye
ICD-10-CM	E10339	Type 1 diabetes mellitus with moderate nonproliferative diabetic retinopathy without macular edema
ICD-10-CM	E103391	Type 1 diabetes mellitus with moderate nonproliferative diabetic retinopathy without macular edema, right eye
ICD-10-CM	E103392	Type 1 diabetes mellitus with moderate nonproliferative diabetic retinopathy without macular edema, left eye
ICD-10-CM	E103393	Type 1 diabetes mellitus with moderate nonproliferative diabetic retinopathy without macular edema, bilateral
ICD-10-CM	E103399	Type 1 diabetes mellitus with moderate nonproliferative diabetic retinopathy without macular edema, unspecified eye
ICD-10-CM	E10341	Type 1 diabetes mellitus with severe nonproliferative diabetic retinopathy with macular edema
ICD-10-CM	E103411	Type 1 diabetes mellitus with severe nonproliferative diabetic retinopathy with macular edema, right eye
ICD-10-CM	E103412	Type 1 diabetes mellitus with severe nonproliferative diabetic retinopathy with macular edema, left eye
ICD-10-CM	E103413	Type 1 diabetes mellitus with severe nonproliferative diabetic retinopathy with macular edema, bilateral
ICD-10-CM	E103419	Type 1 diabetes mellitus with severe nonproliferative diabetic retinopathy with macular edema, unspecified eye
ICD-10-CM	E10349	Type 1 diabetes mellitus with severe nonproliferative diabetic retinopathy without macular edema
ICD-10-CM	E103491	Type 1 diabetes mellitus with severe nonproliferative diabetic retinopathy without macular edema, right eye
ICD-10-CM	E103492	Type 1 diabetes mellitus with severe nonproliferative diabetic retinopathy without macular edema, left eye
ICD-10-CM	E103493	Type 1 diabetes mellitus with severe nonproliferative diabetic retinopathy without macular edema, bilateral
ICD-10-CM	E103499	Type 1 diabetes mellitus with severe nonproliferative diabetic retinopathy without macular edema, unspecified eye
ICD-10-CM	E10351	Type 1 diabetes mellitus with proliferative diabetic retinopathy with macular edema
ICD-10-CM	E103511	Type 1 diabetes mellitus with proliferative diabetic retinopathy with macular edema, right eye
ICD-10-CM	E103512	Type 1 diabetes mellitus with proliferative diabetic retinopathy with macular edema, left eye
ICD-10-CM	E103513	Type 1 diabetes mellitus with proliferative diabetic retinopathy with macular edema, bilateral
ICD-10-CM	E103519	Type 1 diabetes mellitus with proliferative diabetic retinopathy with macular edema, unspecified eye
ICD-10-CM	E103521	Type 1 diabetes mellitus with proliferative diabetic retinopathy with traction retinal detachment involving the macula, right eye
ICD-10-CM	E103522	Type 1 diabetes mellitus with proliferative diabetic retinopathy with traction retinal detachment involving the macula, left eye
ICD-10-CM	E103523	Type 1 diabetes mellitus with proliferative diabetic retinopathy with traction retinal detachment involving the macula, bilateral
ICD-10-CM	E103529	Type 1 diabetes mellitus with proliferative diabetic retinopathy with traction retinal detachment involving the macula, unspecified eye
ICD-10-CM	E103531	Type 1 diabetes mellitus with proliferative diabetic retinopathy with traction retinal detachment not involving the macula, right eye
ICD-10-CM	E103532	Type 1 diabetes mellitus with proliferative diabetic retinopathy with traction retinal detachment not involving the macula, left eye
ICD-10-CM	E103533	Type 1 diabetes mellitus with proliferative diabetic retinopathy with traction retinal detachment not involving the macula, bilateral
ICD-10-CM	E103539	Type 1 diabetes mellitus with proliferative diabetic retinopathy with traction retinal detachment not involving the macula, unspecified eye
ICD-10-CM	E103541	Type 1 diabetes mellitus with proliferative diabetic retinopathy with combined traction retinal detachment and rhegmatogenous retinal detachment, right eye
ICD-10-CM	E103542	Type 1 diabetes mellitus with proliferative diabetic retinopathy with combined traction retinal detachment and rhegmatogenous retinal detachment, left eye
ICD-10-CM	E103543	Type 1 diabetes mellitus with proliferative diabetic retinopathy with combined traction retinal detachment and rhegmatogenous retinal detachment, bilateral
ICD-10-CM	E103549	Type 1 diabetes mellitus with proliferative diabetic retinopathy with combined traction retinal detachment and rhegmatogenous retinal detachment, unspecified eye
ICD-10-CM	E103551	Type 1 diabetes mellitus with stable proliferative diabetic retinopathy, right eye
ICD-10-CM	E103552	Type 1 diabetes mellitus with stable proliferative diabetic retinopathy, left eye
ICD-10-CM	E103553	Type 1 diabetes mellitus with stable proliferative diabetic retinopathy, bilateral
ICD-10-CM	E103559	Type 1 diabetes mellitus with stable proliferative diabetic retinopathy, unspecified eye
ICD-10-CM	E10359	Type 1 diabetes mellitus with proliferative diabetic retinopathy without macular edema
ICD-10-CM	E103591	Type 1 diabetes mellitus with proliferative diabetic retinopathy without macular edema, right eye
ICD-10-CM	E103592	Type 1 diabetes mellitus with proliferative diabetic retinopathy without macular edema, left eye
ICD-10-CM	E103593	Type 1 diabetes mellitus with proliferative diabetic retinopathy without macular edema, bilateral
ICD-10-CM	E103599	Type 1 diabetes mellitus with proliferative diabetic retinopathy without macular edema, unspecified eye
ICD-10-CM	E1036	Type 1 diabetes mellitus with diabetic cataract
ICD-10-CM	E1037X1	Type 1 diabetes mellitus with diabetic macular edema, resolved following treatment, right eye
ICD-10-CM	E1037X2	Type 1 diabetes mellitus with diabetic macular edema, resolved following treatment, left eye
ICD-10-CM	E1037X3	Type 1 diabetes mellitus with diabetic macular edema, resolved following treatment, bilateral
ICD-10-CM	E1037X9	Type 1 diabetes mellitus with diabetic macular edema, resolved following treatment, unspecified eye
ICD-10-CM	E1039	Type 1 diabetes mellitus with other diabetic ophthalmic complication
ICD-10-CM	E1040	Type 1 diabetes mellitus with diabetic neuropathy, unspecified
ICD-10-CM	E1041	Type 1 diabetes mellitus with diabetic mononeuropathy
ICD-10-CM	E1042	Type 1 diabetes mellitus with diabetic polyneuropathy
ICD-10-CM	E1043	Type 1 diabetes mellitus with diabetic autonomic (poly)neuropathy
ICD-10-CM	E1044	Type 1 diabetes mellitus with diabetic amyotrophy
ICD-10-CM	E1049	Type 1 diabetes mellitus with other diabetic neurological complication
ICD-10-CM	E1051	Type 1 diabetes mellitus with diabetic peripheral angiopathy without gangrene
ICD-10-CM	E1052	Type 1 diabetes mellitus with diabetic peripheral angiopathy with gangrene
ICD-10-CM	E1059	Type 1 diabetes mellitus with other circulatory complications
ICD-10-CM	E10610	Type 1 diabetes mellitus with diabetic neuropathic arthropathy
ICD-10-CM	E10618	Type 1 diabetes mellitus with other diabetic arthropathy
ICD-10-CM	E10620	Type 1 diabetes mellitus with diabetic dermatitis
ICD-10-CM	E10621	Type 1 diabetes mellitus with foot ulcer
ICD-10-CM	E10622	Type 1 diabetes mellitus with other skin ulcer
ICD-10-CM	E10628	Type 1 diabetes mellitus with other skin complications
ICD-10-CM	E10630	Type 1 diabetes mellitus with periodontal disease
ICD-10-CM	E10638	Type 1 diabetes mellitus with other oral complications
ICD-10-CM	E10641	Type 1 diabetes mellitus with hypoglycemia with coma
ICD-10-CM	E10649	Type 1 diabetes mellitus with hypoglycemia without coma
ICD-10-CM	E1065	Type 1 diabetes mellitus with hyperglycemia
ICD-10-CM	E1069	Type 1 diabetes mellitus with other specified complication
ICD-10-CM	E108	Type 1 diabetes mellitus with unspecified complications
ICD-10-CM	E109	Type 1 diabetes mellitus without complications
ICD-10-CM	O24011	Pre-existing type 1 diabetes mellitus, in pregnancy, first trimester
ICD-10-CM	O24012	Pre-existing type 1 diabetes mellitus, in pregnancy, second trimester

Exhibit A4. ICD-10-CM Diagnosis Codes for Type 1 Diabetes

Code Type	Code	Description
ICD-10-CM	O24013	Pre-existing type 1 diabetes mellitus, in pregnancy, third trimester
ICD-10-CM	O24019	Pre-existing type 1 diabetes mellitus, in pregnancy, unspecified trimester
ICD-10-CM	O2402	Pre-existing type 1 diabetes mellitus, in childbirth
ICD-10-CM	O2403	Pre-existing type 1 diabetes mellitus, in the puerperium

Exhibit A5. NDC Codes for T2DM Prescription Drugs Used to Exclude Patients from the T1DM Population

NDCs were generated for each of the drug, description, and use categories listed below. The full list of NDCs is available from the authors.
The use of GLP-1s to identify non-T1DM patients may need to be revisited if more recent data is analyzed,due to expanded GLP-1 indicaitons.

Code Type	Drug Category	Description	Diabetes Patient Identification Use
NDC	AGI	Miglitol Tab 25 MG	Hypoglycemics/Antihyperglycemics
NDC	AGI	Miglitol Tab 50 MG	Hypoglycemics/Antihyperglycemics
NDC	AGI	Miglitol Tab 100 MG	Hypoglycemics/Antihyperglycemics
NDC	AGI	Acarbose Tab 50 MG	Hypoglycemics/Antihyperglycemics
NDC	AGI	Acarbose Tab 100 MG	Hypoglycemics/Antihyperglycemics
NDC	AGI	Acarbose Tab 25 MG	Hypoglycemics/Antihyperglycemics
NDC	DPP-4	Saxagliptin HCl Tab 2.5 MG (Base Equiv)	Hypoglycemics/Antihyperglycemics
NDC	DPP-4	Saxagliptin HCl Tab 5 MG (Base Equiv)	Hypoglycemics/Antihyperglycemics
NDC	DPP-4	Sitagliptin Phosphate Tab 50 MG (Base Equiv)	Hypoglycemics/Antihyperglycemics
NDC	DPP-4	Sitagliptin Phosphate Tab 25 MG (Base Equiv)	Hypoglycemics/Antihyperglycemics
NDC	DPP-4	Sitagliptin Phosphate Tab 100 MG (Base Equiv)	Hypoglycemics/Antihyperglycemics
NDC	DPP-4	Linagliptin Tab 5 MG	Hypoglycemics/Antihyperglycemics
NDC	DPP-4	Alogliptin Benzoate Tab 6.25 MG (Base Equiv)	Hypoglycemics/Antihyperglycemics
NDC	DPP-4	Alogliptin Benzoate Tab 12.5 MG (Base Equiv)	Hypoglycemics/Antihyperglycemics
NDC	DPP-4	Alogliptin Benzoate Tab 25 MG (Base Equiv)	Hypoglycemics/Antihyperglycemics
NDC	DPP-4-Metformin	Saxagliptin-Metformin HCl Tab SR 24HR 5-500 MG	Hypoglycemics/Antihyperglycemics
NDC	DPP-4-Metformin	Saxagliptin-Metformin HCl Tab SR 24HR 2.5-1000 MG	Hypoglycemics/Antihyperglycemics
NDC	DPP-4-Metformin	Saxagliptin-Metformin HCl Tab SR 24HR 5-1000 MG	Hypoglycemics/Antihyperglycemics
NDC	DPP-4-Metformin	Sitagliptin-Metformin HCl Tab SR 24HR 50-500 MG	Hypoglycemics/Antihyperglycemics
NDC	DPP-4-Metformin	Sitagliptin-Metformin HCl Tab ER 24HR 50-1000 MG	Hypoglycemics/Antihyperglycemics
NDC	DPP-4-Metformin	Sitagliptin-Metformin HCl Tab SR 24HR 50-1000 MG	Hypoglycemics/Antihyperglycemics
NDC	DPP-4-Metformin	Sitagliptin-Metformin HCl Tab ER 24HR 100-1000 MG	Hypoglycemics/Antihyperglycemics
NDC	DPP-4-Metformin	Sitagliptin-Metformin HCl Tab SR 24HR 100-1000 MG	Hypoglycemics/Antihyperglycemics
NDC	DPP-4-Metformin	Sitagliptin-Metformin HCl Tab 50-500 MG	Hypoglycemics/Antihyperglycemics
NDC	DPP-4-Metformin	Sitagliptin-Metformin HCl Tab 50-1000 MG	Hypoglycemics/Antihyperglycemics
NDC	DPP-4-Metformin	Linagliptin-Metformin HCl Tab 2.5-500 MG	Hypoglycemics/Antihyperglycemics
NDC	DPP-4-Metformin	Linagliptin-Metformin HCl Tab 2.5-850 MG	Hypoglycemics/Antihyperglycemics
NDC	DPP-4-Metformin	Linagliptin-Metformin HCl Tab 2.5-1000 MG	Hypoglycemics/Antihyperglycemics
NDC	DPP-4-Metformin	Linagliptin-Metformin HCl Tab SR 24HR 2.5-1000 MG	Hypoglycemics/Antihyperglycemics
NDC	DPP-4-Metformin	Linagliptin-Metformin HCl Tab SR 24HR 5-1000 MG	Hypoglycemics/Antihyperglycemics
NDC	DPP-4-Metformin	Alogliptin-Metformin HCl Tab 12.5-500 MG	Hypoglycemics/Antihyperglycemics
NDC	DPP-4-Metformin	Alogliptin-Metformin HCl Tab 12.5-1000 MG	Hypoglycemics/Antihyperglycemics
NDC	DPP-4-Thiazolidinedione	Alogliptin-Pioglitazone Tab 12.5-15 MG	Hypoglycemics/Antihyperglycemics
NDC	DPP-4-Thiazolidinedione	Alogliptin-Pioglitazone Tab 12.5-30 MG	Hypoglycemics/Antihyperglycemics
NDC	DPP-4-Thiazolidinedione	Alogliptin-Pioglitazone Tab 12.5-45 MG	Hypoglycemics/Antihyperglycemics
NDC	DPP-4-Thiazolidinedione	Alogliptin-Pioglitazone Tab 25-15 MG	Hypoglycemics/Antihyperglycemics
NDC	DPP-4-Thiazolidinedione	Alogliptin-Pioglitazone Tab 25-30 MG	Hypoglycemics/Antihyperglycemics
NDC	DPP-4-Thiazolidinedione	Alogliptin-Pioglitazone Tab 25-45 MG	Hypoglycemics/Antihyperglycemics
NDC	DPP-4-HMG-CoA Inhibitor	Sitagliptin-Simvastatin Tab 50-10 MG	Hypoglycemics/Antihyperglycemics
NDC	DPP-4-HMG-CoA Inhibitor	Sitagliptin-Simvastatin Tab 50-20 MG	Hypoglycemics/Antihyperglycemics
NDC	DPP-4-HMG-CoA Inhibitor	Sitagliptin-Simvastatin Tab 50-40 MG	Hypoglycemics/Antihyperglycemics
NDC	DPP-4-HMG-CoA Inhibitor	Sitagliptin-Simvastatin Tab 100-10 MG	Hypoglycemics/Antihyperglycemics
NDC	DPP-4-HMG-CoA Inhibitor	Sitagliptin-Simvastatin Tab 100-20 MG	Hypoglycemics/Antihyperglycemics
NDC	DPP-4-HMG-CoA Inhibitor	Sitagliptin-Simvastatin Tab 100-40 MG	Hypoglycemics/Antihyperglycemics
NDC	Glinide	Nateglinide Tab 60 MG	Hypoglycemics/Antihyperglycemics
NDC	Glinide	Nateglinide Tab 120 MG	Hypoglycemics/Antihyperglycemics
NDC	Glinide	Repaglinide Tab 0.5 MG	Hypoglycemics/Antihyperglycemics
NDC	Glinide	Repaglinide Tab 1 MG	Hypoglycemics/Antihyperglycemics
NDC	Glinide	Repaglinide Tab 2 MG	Hypoglycemics/Antihyperglycemics
NDC	Glinide-Metformin	Repaglinide-Metformin HCl Tab 2-500 MG	Hypoglycemics/Antihyperglycemics
NDC	Glinide-Metformin	Repaglinide-Metformin HCl Tab 1-500 MG	Hypoglycemics/Antihyperglycemics
NDC	GLP-1	Dulaglutide Soln Pen-injector 0.75 MG/0.5ML	Hypoglycemics/Antihyperglycemics
NDC	GLP-1	Dulaglutide Soln Pen-injector 1.5 MG/0.5ML	Hypoglycemics/Antihyperglycemics
NDC	GLP-1	Dulaglutide Soln Pen-injector 3 MG/0.5ML	Hypoglycemics/Antihyperglycemics
NDC	GLP-1	Dulaglutide Soln Pen-injector 4.5 MG/0.5ML	Hypoglycemics/Antihyperglycemics
NDC	GLP-1	Lixisenatide Soln Pen-injector 20 MCG/0.2ML (100 MCG/ML)	Hypoglycemics/Antihyperglycemics
NDC	GLP-1	Lixisenatide Pen-inj Starter Kit 10 MCG/0.2ML & 20 MCG/0.2ML	Hypoglycemics/Antihyperglycemics
NDC	GLP-1	Liraglutide (Weight Mngmt) Soln Pen-Inj 18 MG/3ML (6 MG/ML)	Hypoglycemics/Antihyperglycemics
NDC	GLP-1	Liraglutide Soln Pen-injector 18 MG/3ML (6 MG/ML)	Hypoglycemics/Antihyperglycemics
NDC	GLP-1	Semaglutide Soln Pen-inj 0.25 or 0.5 MG/DOSE (2 MG/1.5ML)	Hypoglycemics/Antihyperglycemics
NDC	GLP-1	Semaglutide Soln Pen-inj 1 MG/DOSE (2 MG/1.5ML)	Hypoglycemics/Antihyperglycemics
NDC	GLP-1	Semaglutide Tab 3 MG	Hypoglycemics/Antihyperglycemics
NDC	GLP-1	Semaglutide Tab 7 MG	Hypoglycemics/Antihyperglycemics
NDC	GLP-1	Semaglutide Tab 14 MG	Hypoglycemics/Antihyperglycemics
NDC	GLP-1	Albiglutide For Soln Pen-injector 30 MG	Hypoglycemics/Antihyperglycemics
NDC	GLP-1	Albiglutide For Soln Pen-injector 50 MG	Hypoglycemics/Antihyperglycemics
NDC	GLP-1	Exenatide Soln Pen-injector 5 MCG/0.02ML	Hypoglycemics/Antihyperglycemics
NDC	GLP-1	Exenatide Extended Release for Inj Susp 2 MG	Hypoglycemics/Antihyperglycemics
NDC	GLP-1	Exenatide Soln Pen-injector 10 MCG/0.04ML	Hypoglycemics/Antihyperglycemics
NDC	GLP-1	Exenatide Extended Release for Susp Pen-injector 2 MG	Hypoglycemics/Antihyperglycemics
NDC	GLP-1	Exenatide Extended Release Susp Auto-Injector 2 MG/0.85ML	Hypoglycemics/Antihyperglycemics
NDC	GLP-1	Exenatide Inj 10 MCG/0.04ML	Hypoglycemics/Antihyperglycemics
NDC	GLP-1	Exenatide 2 mg subcutaneous injection, extended release	Hypoglycemics/Antihyperglycemics
NDC	SGLT2	Dapagliflozin Propanediol Tab 5 MG (Base Equivalent)	Hypoglycemics/Antihyperglycemics
NDC	SGLT2	Dapagliflozin Propanediol Tab 10 MG (Base Equivalent)	Hypoglycemics/Antihyperglycemics
NDC	SGLT2	Ertugliflozin L-Pyroglutamic Acid Tab 5 MG (Base Equiv)	Hypoglycemics/Antihyperglycemics
NDC	SGLT2	Ertugliflozin L-Pyroglutamic Acid Tab 15 MG (Base Equiv)	Hypoglycemics/Antihyperglycemics

Exhibit A5. NDC Codes for T2DM Prescription Drugs Used to Exclude Patients from the T1DM Population

NDCs were generated for each of the drug, description, and use categories listed below. The full list of NDCs is available from the authors.
The use of GLP-1s to identify non-T1DM patients may need to be revisited if more recent data is analyzed,due to expanded GLP-1 indicaitons.

Code Type	Drug Category	Description	Diabetes Patient Identification Use
NDC	AGI	Miglitol Tab 25 MG	Hypoglycemics/Antihyperglycemics
NDC	SGLT2	Empagliflozin Tab 10 MG	Hypoglycemics/Antihyperglycemics
NDC	SGLT2	Empagliflozin Tab 25 MG	Hypoglycemics/Antihyperglycemics
NDC	SGLT2	Canagliflozin Tab 100 MG	Hypoglycemics/Antihyperglycemics
NDC	SGLT2	Canagliflozin Tab 300 MG	Hypoglycemics/Antihyperglycemics
NDC	SGLT2-DPP-4	Ertugliflozin-Sitagliptin Tab 5-100 MG	Hypoglycemics/Antihyperglycemics
NDC	SGLT2-DPP-4	Ertugliflozin-Sitagliptin Tab 15-100 MG	Hypoglycemics/Antihyperglycemics
NDC	SGLT2-DPP-4	Dapagliflozin-Saxagliptin Tab 5-5 MG	Hypoglycemics/Antihyperglycemics
NDC	SGLT2-DPP-4	Dapagliflozin-Saxagliptin Tab 10-5 MG	Hypoglycemics/Antihyperglycemics
NDC	SGLT2-DPP-4	Empagliflozin-Linagliptin Tab 25-5 MG	Hypoglycemics/Antihyperglycemics
NDC	SGLT2-DPP-4	Empagliflozin-Linagliptin Tab 10-5 MG	Hypoglycemics/Antihyperglycemics
NDC	SGLT2-Metformin	Ertugliflozin-Metformin HCl Tab 2.5-500 MG	Hypoglycemics/Antihyperglycemics
NDC	SGLT2-Metformin	Ertugliflozin-Metformin HCl Tab 7.5-500 MG	Hypoglycemics/Antihyperglycemics
NDC	SGLT2-Metformin	Ertugliflozin-Metformin HCl Tab 2.5-1000 MG	Hypoglycemics/Antihyperglycemics
NDC	SGLT2-Metformin	Ertugliflozin-Metformin HCl Tab 7.5-1000 MG	Hypoglycemics/Antihyperglycemics
NDC	SGLT2-Metformin	Dapagliflozin-Metformin HCl Tab ER 24HR 2.5-1000 MG	Hypoglycemics/Antihyperglycemics
NDC	SGLT2-Metformin	Dapagliflozin-Metformin HCl Tab SR 24HR 5-500 MG	Hypoglycemics/Antihyperglycemics
NDC	SGLT2-Metformin	Dapagliflozin-Metformin HCl Tab SR 24HR 5-1000 MG	Hypoglycemics/Antihyperglycemics
NDC	SGLT2-Metformin	Dapagliflozin-Metformin HCl Tab SR 24HR 10-500 MG	Hypoglycemics/Antihyperglycemics
NDC	SGLT2-Metformin	Dapagliflozin-Metformin HCl Tab SR 24HR 10-1000 MG	Hypoglycemics/Antihyperglycemics
NDC	SGLT2-Metformin	Empagliflozin-Metformin HCl Tab 5-500 MG	Hypoglycemics/Antihyperglycemics
NDC	SGLT2-Metformin	Empagliflozin-Metformin HCl Tab 12.5-1000 MG	Hypoglycemics/Antihyperglycemics
NDC	SGLT2-Metformin	Empagliflozin-Metformin HCl Tab 5-1000 MG	Hypoglycemics/Antihyperglycemics
NDC	SGLT2-Metformin	Empagliflozin-Metformin HCl Tab 12.5-500 MG	Hypoglycemics/Antihyperglycemics
NDC	SGLT2-Metformin	Empagliflozin-Metformin HCl Tab SR 24HR 10-1000 MG	Hypoglycemics/Antihyperglycemics
NDC	SGLT2-Metformin	Empagliflozin-Metformin HCl Tab SR 24HR 5-1000 MG	Hypoglycemics/Antihyperglycemics
NDC	SGLT2-Metformin	Empagliflozin-Metformin HCl Tab SR 24HR 25-1000 MG	Hypoglycemics/Antihyperglycemics
NDC	SGLT2-Metformin	Empagliflozin-Metformin HCl Tab SR 24HR 12.5-1000 MG	Hypoglycemics/Antihyperglycemics
NDC	SGLT2-Metformin	Canagliflozin-Metformin HCl Tab 50-500 MG	Hypoglycemics/Antihyperglycemics
NDC	SGLT2-Metformin	Canagliflozin-Metformin HCl Tab 50-1000 MG	Hypoglycemics/Antihyperglycemics
NDC	SGLT2-Metformin	Canagliflozin-Metformin HCl Tab 150-500 MG	Hypoglycemics/Antihyperglycemics
NDC	SGLT2-Metformin	Canagliflozin-Metformin HCl Tab 150-1000 MG	Hypoglycemics/Antihyperglycemics
NDC	SGLT2-Metformin	Canagliflozin-Metformin HCl Tab SR 24HR 50-500 MG	Hypoglycemics/Antihyperglycemics
NDC	SGLT2-Metformin	Canagliflozin-Metformin HCl Tab SR 24HR 50-1000 MG	Hypoglycemics/Antihyperglycemics
NDC	SGLT2-Metformin	Canagliflozin-Metformin HCl Tab SR 24HR 150-500 MG	Hypoglycemics/Antihyperglycemics
NDC	SGLT2-Metformin	Canagliflozin-Metformin HCl Tab SR 24HR 150-1000 MG	Hypoglycemics/Antihyperglycemics
NDC	Sulfonylurea	Glyburide Micronized Tab 1.5 MG	Hypoglycemics/Antihyperglycemics
NDC	Sulfonylurea	Glyburide Micronized Tab 3 MG	Hypoglycemics/Antihyperglycemics
NDC	Sulfonylurea	Glyburide Micronized Tab 6 MG	Hypoglycemics/Antihyperglycemics
NDC	Sulfonylurea	Glyburide Tab 2.5 MG	Hypoglycemics/Antihyperglycemics
NDC	Sulfonylurea	Glyburide Tab 5 MG	Hypoglycemics/Antihyperglycemics
NDC	Sulfonylurea	Glyburide Tab 1.25 MG	Hypoglycemics/Antihyperglycemics
NDC	Sulfonylurea	Glimepiride Tab 1 MG	Hypoglycemics/Antihyperglycemics
NDC	Sulfonylurea	Glimepiride Tab 2 MG	Hypoglycemics/Antihyperglycemics
NDC	Sulfonylurea	Glimepiride Tab 4 MG	Hypoglycemics/Antihyperglycemics
NDC	Sulfonylurea	Glipizide Tab SR 24HR 2.5 MG	Hypoglycemics/Antihyperglycemics
NDC	Sulfonylurea	Glipizide Tab SR 24HR 5 MG	Hypoglycemics/Antihyperglycemics
NDC	Sulfonylurea	Glipizide Tab SR 24HR 10 MG	Hypoglycemics/Antihyperglycemics
NDC	Sulfonylurea	Glipizide Tab 5 MG	Hypoglycemics/Antihyperglycemics
NDC	Sulfonylurea	Glipizide Tab 10 MG	Hypoglycemics/Antihyperglycemics
NDC	Sulfonylurea	Glyburide 2.5 mg oral tablet	Hypoglycemics/Antihyperglycemics
NDC	Sulfonylurea	Glipizide 5 mg oral tablet	Hypoglycemics/Antihyperglycemics
NDC	Sulfonylurea	Chlorpropamide Tab 250 MG	Hypoglycemics/Antihyperglycemics
NDC	Sulfonylurea	Glyburide 5 mg oral tablet	Hypoglycemics/Antihyperglycemics
NDC	Sulfonylurea	Glipizide 10 mg oral tablet	Hypoglycemics/Antihyperglycemics
NDC	Sulfonylurea	Glipizide 10 mg oral tablet, extended release	Hypoglycemics/Antihyperglycemics
NDC	Sulfonylurea	Glipizide 5 mg oral tablet, extended release	Hypoglycemics/Antihyperglycemics
NDC	Sulfonylurea	Glyburide 1.25 mg oral tablet	Hypoglycemics/Antihyperglycemics
NDC	Sulfonylurea	Glimepiride 1 mg oral tablet	Hypoglycemics/Antihyperglycemics
NDC	Sulfonylurea	Glimepiride 2 mg oral tablet	Hypoglycemics/Antihyperglycemics
NDC	Sulfonylurea	Chlorpropamide Tab 100 MG	Hypoglycemics/Antihyperglycemics
NDC	Sulfonylurea	Tolbutamide Tab 500 MG	Hypoglycemics/Antihyperglycemics
NDC	Sulfonylurea	Tolazamide Tab 250 MG	Hypoglycemics/Antihyperglycemics
NDC	Sulfonylurea	Tolazamide Tab 500 MG	Hypoglycemics/Antihyperglycemics
NDC	Sulfonylurea	Glipizide Tab ER 24HR 2.5 MG	Hypoglycemics/Antihyperglycemics
NDC	Sulfonylurea	Glipizide Tab ER 24HR 5 MG	Hypoglycemics/Antihyperglycemics
NDC	Sulfonylurea	Glipizide Tab ER 24HR 10 MG	Hypoglycemics/Antihyperglycemics
NDC	Sulfonylurea	Glipizide Powder	Hypoglycemics/Antihyperglycemics
NDC	Sulfonylurea	Glyburide Powder	Hypoglycemics/Antihyperglycemics
NDC	Sulfonylurea	Chlorpropamid 250 mg oral tablet	Hypoglycemics/Antihyperglycemics
NDC	Sulfonylurea	Glimepiride 4 mg oral tablet	Hypoglycemics/Antihyperglycemics
NDC	Sulfonylurea	Glyburide Micronized Tab 4.5 MG	Hypoglycemics/Antihyperglycemics
NDC	Sulfonylurea	Glyburide micronized 1.5 mg oral tablet	Hypoglycemics/Antihyperglycemics
NDC	Sulfonylurea	Glyburide Tab 1.25 MG	Hypoglycemics/Antihyperglycemics
NDC	Sulfonylurea-Metformin	Glyburide-Metformin Tab 1.25-250 MG	Hypoglycemics/Antihyperglycemics
NDC	Sulfonylurea-Metformin	Glyburide-Metformin Tab 2.5-500 MG	Hypoglycemics/Antihyperglycemics
NDC	Sulfonylurea-Metformin	Glyburide-Metformin Tab 5-500 MG	Hypoglycemics/Antihyperglycemics
NDC	Sulfonylurea-Metformin	Glipizide-Metformin HCl Tab 2.5-500 MG	Hypoglycemics/Antihyperglycemics

Exhibit A5. NDC Codes for T2DM Prescription Drugs Used to Exclude Patients from the T1DM Population

NDCs were generated for each of the drug, description, and use categories listed below. The full list of NDCs is available from the authors.
The use of GLP-1s to identify non-T1DM patients may need to be revisited if more recent data is analyzed,due to expanded GLP-1 indicaitons.

Code Type	Drug Category	Description	Diabetes Patient Identification Use
NDC	AGI	Miglitol Tab 25 MG	Hypoglycemics/Antihyperglycemics
NDC	Sulfonylurea-Metformin	Glipizide-Metformin HCl Tab 5-500 MG	Hypoglycemics/Antihyperglycemics
NDC	Sulfonylurea-Metformin	Glipizide-Metformin HCl Tab 2.5-250 MG	Hypoglycemics/Antihyperglycemics
NDC	Sulfonylurea-Metformin	Glyburide-metformin 1.25 mg-250 mg oral tablet	Hypoglycemics/Antihyperglycemics
NDC	Sulfonylurea-Metformin	Glyburide-metformin 2.5 mg-500 mg oral tablet	Hypoglycemics/Antihyperglycemics
NDC	Sulfonylurea-Metformin	Glyburide-metformin 5 mg-500 mg oral tablet	Hypoglycemics/Antihyperglycemics
NDC	Sulfonylurea-Metformin	Glyburide Micronized Tab 1.5 MG	Hypoglycemics/Antihyperglycemics
NDC	Sulfonylurea-Thiazolidinedione	Rosiglitazone Maleate-Glimepiride Tab 8-2 MG	Hypoglycemics/Antihyperglycemics
NDC	Sulfonylurea-Thiazolidinedione	Rosiglitazone Maleate-Glimepiride Tab 8-4 MG	Hypoglycemics/Antihyperglycemics
NDC	Sulfonylurea-Thiazolidinedione	Rosiglitazone Maleate-Glimepiride Tab 4-1 MG	Hypoglycemics/Antihyperglycemics
NDC	Sulfonylurea-Thiazolidinedione	Rosiglitazone Maleate-Glimepiride Tab 4-2 MG	Hypoglycemics/Antihyperglycemics
NDC	Sulfonylurea-Thiazolidinedione	Rosiglitazone Maleate-Glimepiride Tab 4-4 MG	Hypoglycemics/Antihyperglycemics
NDC	Sulfonylurea-Thiazolidinedione	Pioglitazone HCl-Glimepiride Tab 30-2 MG	Hypoglycemics/Antihyperglycemics
NDC	Sulfonylurea-Thiazolidinedione	Pioglitazone HCl-Glimepiride Tab 30-4 MG	Hypoglycemics/Antihyperglycemics
NDC	Thiazolidinedione	Rosiglitazone Maleate Tab 2 MG (Base Equiv)	Hypoglycemics/Antihyperglycemics
NDC	Thiazolidinedione	Rosiglitazone Maleate Tab 4 MG (Base Equiv)	Hypoglycemics/Antihyperglycemics
NDC	Thiazolidinedione	Rosiglitazone Maleate Tab 8 MG (Base Equiv)	Hypoglycemics/Antihyperglycemics
NDC	Thiazolidinedione	Pioglitazone HCl Tab 45 MG (Base Equiv)	Hypoglycemics/Antihyperglycemics
NDC	Thiazolidinedione	Pioglitazone HCl Tab 30 MG (Base Equiv)	Hypoglycemics/Antihyperglycemics
NDC	Thiazolidinedione	Pioglitazone HCl Tab 15 MG (Base Equiv)	Hypoglycemics/Antihyperglycemics
NDC	Thiazolidinedione	Pioglitazone 45 mg oral tablet	Hypoglycemics/Antihyperglycemics
NDC	Thiazolidinedione	Rosiglitazone 4 mg oral tablet	Hypoglycemics/Antihyperglycemics
NDC	Thiazolidinedione	Rosiglitazone 8 mg oral tablet	Hypoglycemics/Antihyperglycemics
NDC	Thiazolidinedione	Pioglitazone 30 mg oral tablet	Hypoglycemics/Antihyperglycemics
NDC	Thiazolidinedione	Pioglitazone 15 mg oral tablet	Hypoglycemics/Antihyperglycemics
NDC	Thiazolidinedione	Rosiglitazone Maleate-Metformin HCl Tab 2-500 MG	Hypoglycemics/Antihyperglycemics
NDC	Thiazolidinedione-Metformin	Rosiglitazone Maleate-Metformin HCl Tab 2-1000 MG	Hypoglycemics/Antihyperglycemics
NDC	Thiazolidinedione-Metformin	Rosiglitazone Maleate-Metformin HCl Tab 4-1000 MG	Hypoglycemics/Antihyperglycemics
NDC	Thiazolidinedione-Metformin	Rosiglitazone Maleate-Metformin HCl Tab 2-500 MG	Hypoglycemics/Antihyperglycemics
NDC	Thiazolidinedione-Metformin	Rosiglitazone Maleate-Metformin HCl Tab 4-500 MG	Hypoglycemics/Antihyperglycemics
NDC	Thiazolidinedione-Metformin	Pioglitazone HCl-Metformin HCl Tab 15-500 MG	Hypoglycemics/Antihyperglycemics
NDC	Thiazolidinedione-Metformin	Pioglitazone HCl-Metformin HCl Tab 15-850 MG	Hypoglycemics/Antihyperglycemics
NDC	Thiazolidinedione-Metformin	Pioglitazone HCl-Metformin HCl Tab SR 24HR 30-1000 MG	Hypoglycemics/Antihyperglycemics
NDC	Thiazolidinedione-Metformin	Pioglitazone HCl-Metformin HCl Tab SR 24HR 15-1000 MG	Hypoglycemics/Antihyperglycemics

Exhibit A6. State Region Mapping

State	Mapped Region
Alaska	West
Alabama	South
Arkansas	South
Arizona	West
California	West
Colorado	West
Connecticut	Northeast
District of Columbia	South
Delaware	South
Florida	South
Georgia	South
Hawaii	West
Iowa	Midwest
Idaho	West
Illinois	Midwest
Indiana	Midwest
Kansas	Midwest
Kentucky	South
Louisiana	South
Massachusetts	Northeast
Maryland	South
Maine	Northeast
Michigan	Midwest
Minnesota	Midwest
Missouri	Midwest
Mississippi	South
Montana	West
North Carolina	South
North Dakota	Midwest
Nebraska	Midwest
New Hampshire	Northeast
New Jersey	Northeast
New Mexico	West
Nevada	West
New York	Northeast
Ohio	Midwest
Oklahoma	South
Oregon	West
Pennsylvania	Northeast
Rhode Island	Northeast
South Carolina	South
South Dakota	Midwest
Tennessee	South
Texas	South
Utah	West
Virginia	South
Vermont	Northeast
Washington	West
Wisconsin	Midwest
West Virginia	South
Wyoming	West

Exhibit A7. Prevalence and Extrapolation - Total T1DM Population

Description	Source	FFS (a AND/OR b)	MA-PD (c AND/OR D)	Other Medicare Health Plan Enrollment	Commercial (<65)	Medicaid (excluding duals)	VA (18-64)	Uninsured (<65)*
2019 National Enrollment	Literature Review	38,577,012	22,200,000	3,622,988	172,700,000	64,896,849	4,236,144	32,800,000
Annual Prevalence	Claims Analysis	0.867%	0.863%	0.867%	0.488%	0.406%	0.488%	0.406%
Extrapolated to 2019	2019 National Enrollment * Annual Prevalence	334,524	191,498	31,417	843,567	263,710	20,692	133,283

Exhibit A8. Standard Mortality Rates by Age and Sex

CDC WONDER. *Multiple Cause of Death, 1999-2020 Request* .
https://wonder.cdc.gov/mcd.html

Age	M/F	WONDER Population	WONDER Deaths	WONDER Mortality Rate
0	M	1,935,117	11,674	0.603%
1	M	1,958,585	825	0.042%
2	M	2,005,544	515	0.026%
3	M	2,043,010	390	0.019%
4	M	2,066,951	311	0.015%
5	M	2,061,200	291	0.014%
6	M	2,052,956	266	0.013%
7	M	2,055,735	243	0.012%
8	M	2,079,723	251	0.012%
9	M	2,073,148	239	0.012%
10	M	2,071,778	291	0.014%
11	M	2,138,037	324	0.015%
12	M	2,149,819	351	0.016%
13	M	2,132,987	394	0.018%
14	M	2,125,640	536	0.025%
15	M	2,129,720	754	0.035%
16	M	2,116,165	1,040	0.049%
17	M	2,112,553	1,353	0.064%
18	M	2,172,385	1,972	0.091%
19	M	2,214,784	2,242	0.101%
20	M	2,183,139	2,452	0.112%
21	M	2,187,086	2,871	0.131%
22	M	2,195,296	2,909	0.133%
23	M	2,221,758	2,979	0.134%
24	M	2,277,473	3,176	0.139%
25	M	2,325,853	3,481	0.150%
26	M	2,365,886	3,696	0.156%
27	M	2,421,270	3,864	0.160%
28	M	2,451,756	4,129	0.168%
29	M	2,439,805	4,272	0.175%
30	M	2,340,865	4,301	0.184%
31	M	2,281,327	4,282	0.188%
32	M	2,239,886	4,269	0.191%
33	M	2,243,280	4,410	0.197%
34	M	2,249,252	4,647	0.207%
35	M	2,167,622	4,671	0.215%
36	M	2,191,368	4,854	0.222%
37	M	2,181,162	5,066	0.232%
38	M	2,149,359	5,281	0.246%
39	M	2,195,430	5,369	0.245%
40	M	2,046,663	5,628	0.275%
41	M	2,001,502	5,385	0.269%
42	M	1,972,584	5,655	0.287%
43	M	1,913,289	5,558	0.290%
44	M	1,973,101	5,969	0.303%
45	M	1,900,492	6,309	0.332%
46	M	1,919,298	6,937	0.361%
47	M	2,005,221	7,793	0.389%
48	M	2,114,614	8,843	0.418%
49	M	2,145,730	9,476	0.442%
50	M	2,025,376	9,940	0.491%
51	M	1,976,883	10,653	0.539%
52	M	1,968,619	11,605	0.589%
53	M	2,001,325	12,550	0.627%
54	M	2,114,408	14,741	0.697%
55	M	2,137,586	16,534	0.773%
56	M	2,125,123	17,793	0.837%
57	M	2,111,795	19,341	0.916%
58	M	2,125,774	20,908	0.984%
59	M	2,142,211	22,479	1.049%
60	M	2,052,677	23,299	1.135%

Exhibit A8. Standard Mortality Rates by Age and Sex

CDC WONDER. *Multiple Cause of Death, 1999-2020 Request* .
https://wonder.cdc.gov/mcd.html

Age	M/F	WONDER Population	WONDER Deaths	WONDER Mortality Rate
61	M	2,027,951	25,181	1.242%
62	M	1,991,220	26,654	1.339%
63	M	1,905,842	27,524	1.444%
64	M	1,879,040	28,212	1.501%
65	M	1,783,428	29,113	1.632%
66	M	1,703,888	29,767	1.747%
67	M	1,627,863	30,240	1.858%
68	M	1,564,129	30,695	1.962%
69	M	1,520,465	31,123	2.047%
70	M	1,462,120	32,921	2.252%
71	M	1,435,378	34,789	2.424%
72	M	1,483,236	37,706	2.542%
73	M	1,074,771	31,566	2.937%
74	M	1,044,301	32,327	3.096%
75	M	998,390	34,576	3.463%
76	M	1,003,690	37,415	3.728%
77	M	854,930	35,401	4.141%
78	M	760,170	34,513	4.540%
79	M	701,319	34,591	4.932%
80	M	643,638	35,264	5.479%
81	M	593,628	35,972	6.060%
82	M	525,766	35,419	6.737%
83	M	480,165	35,821	7.460%
84	M	436,527	36,128	8.276%
85+	M	2,376,488	338,165	14.230%
0	F	1,847,935	9,247	0.500%
1	F	1,871,014	649	0.035%
2	F	1,916,500	401	0.021%
3	F	1,955,655	320	0.016%
4	F	1,976,372	265	0.013%
5	F	1,967,081	245	0.012%
6	F	1,964,271	224	0.011%
7	F	1,966,584	196	0.010%
8	F	1,986,471	184	0.009%
9	F	1,988,726	194	0.010%
10	F	1,989,162	189	0.010%
11	F	2,051,224	212	0.010%
12	F	2,058,568	250	0.012%
13	F	2,042,234	293	0.014%
14	F	2,038,819	324	0.016%
15	F	2,045,739	383	0.019%
16	F	2,034,255	445	0.022%
17	F	2,029,872	533	0.026%
18	F	2,083,442	760	0.036%
19	F	2,115,655	776	0.037%
20	F	2,086,544	861	0.041%
21	F	2,091,237	950	0.045%
22	F	2,103,476	973	0.046%
23	F	2,119,886	1,167	0.055%
24	F	2,167,045	1,175	0.054%
25	F	2,213,205	1,271	0.057%
26	F	2,245,334	1,390	0.062%
27	F	2,312,599	1,519	0.066%
28	F	2,366,969	1,628	0.069%
29	F	2,366,339	1,719	0.073%
30	F	2,273,519	1,882	0.083%
31	F	2,220,984	1,943	0.087%
32	F	2,181,619	1,994	0.091%
33	F	2,189,693	2,273	0.104%
34	F	2,210,880	2,208	0.100%
35	F	2,148,244	2,327	0.108%
36	F	2,181,076	2,472	0.113%

Exhibit A8. Standard Mortality Rates by Age and Sex

CDC WONDER. *Multiple Cause of Death, 1999-2020 Request* .
https://wonder.cdc.gov/mcd.html

Age	M/F	WONDER Population	WONDER Deaths	WONDER Mortality Rate
37	F	2,180,124	2,626	0.120%
38	F	2,156,217	2,896	0.134%
39	F	2,186,919	3,076	0.141%
40	F	2,058,650	3,011	0.146%
41	F	2,018,752	3,071	0.152%
42	F	2,002,157	3,136	0.157%
43	F	1,940,751	3,370	0.174%
44	F	1,994,174	3,565	0.179%
45	F	1,937,417	3,861	0.199%
46	F	1,970,074	4,261	0.216%
47	F	2,052,817	4,786	0.233%
48	F	2,168,043	5,542	0.256%
49	F	2,184,045	5,931	0.272%
50	F	2,071,196	6,211	0.300%
51	F	2,027,460	6,545	0.323%
52	F	2,033,163	7,183	0.353%
53	F	2,067,526	8,043	0.389%
54	F	2,191,195	9,183	0.419%
55	F	2,236,979	10,259	0.459%
56	F	2,235,893	11,173	0.500%
57	F	2,230,590	12,317	0.552%
58	F	2,259,796	13,439	0.595%
59	F	2,271,644	14,210	0.626%
60	F	2,199,986	15,391	0.700%
61	F	2,187,221	16,341	0.747%
62	F	2,165,425	17,321	0.800%
63	F	2,090,246	17,837	0.853%
64	F	2,071,538	18,724	0.904%
65	F	1,991,169	19,046	0.957%
66	F	1,914,181	19,793	1.034%
67	F	1,836,574	20,479	1.115%
68	F	1,781,346	21,517	1.208%
69	F	1,731,958	22,639	1.307%
70	F	1,674,584	24,422	1.458%
71	F	1,647,705	26,607	1.615%
72	F	1,707,812	29,424	1.723%
73	F	1,259,662	25,287	2.007%
74	F	1,238,863	26,098	2.107%
75	F	1,199,896	28,571	2.381%
76	F	1,218,702	31,604	2.593%
77	F	1,056,331	30,493	2.887%
78	F	960,647	30,611	3.186%
79	F	898,590	31,718	3.530%
80	F	831,640	33,085	3.978%
81	F	788,013	34,649	4.397%
82	F	715,575	35,705	4.990%
83	F	671,025	37,703	5.619%
84	F	631,230	38,788	6.145%
85+	F	4,228,470	535,581	12.666%

Exhibit A9. Moratlity Load Development

	A	B	C	D = B/C	E = D/Yellow Cell
**Ages 0-64**	**Beneficiaries Observed in 2020 Data**	**Deaths Observed in 2020 Data**	**Deaths in population, assuming mortality follows that of a standard mortality table**	**Ratio of Deaths in Data to Deaths under Standard Mortality**	**Ratio / Denom Ratio (Covid Adjustment)**
Denominator	2,891,098	88,961	16,802	5.295	1.000
T1DM	57,146	5,084	323	15.740	2.973

**Ages 65+**	**Beneficiaries Observed in 2020 Data**	**Deaths Observed in 2020 Data**	**Deaths in population, assuming mortality follows that of a standard mortality table**	**Ratio of Deaths in Data to Deaths under Standard Mortality**	**Ratio / Denom Ratio (Covid Adjustment)**
Denominator	17,716,452	904,081	727,555	1.243	1.000
T1DM	121,554	13,512	4,356	3.102	2.496

Exhibit A10. CDC WONDER Demographic Projections

CDC WONDER provides ten-year population projections from 2020 to 2030. The sample data below shows the projections by age, sex, ethnicity, and race.
We used the age, sex, and race specific projections to support our modeling projections over the 10 year period.
http://wonder.cdc.gov/wonder/help/PopulationProjections.html

Age	Age Code	Gender	Gender Code	Ethnicity	Ethnicity Code	Race	Race Code	Year	Year Code	Projected Populations
< 1 year	0	Female	F	Hispanic	2135-2	American Indian or Alaska Native	1002-5	2020	2020	14,408
< 1 year	0	Female	F	Hispanic	2135-2	American Indian or Alaska Native	1002-5	2030	2030	15,506
< 1 year	0	Female	F	Hispanic	2135-2	Asian	2028-9	2020	2020	5,172
< 1 year	0	Female	F	Hispanic	2135-2	Asian	2028-9	2030	2030	5,871
< 1 year	0	Female	F	Hispanic	2135-2	Black or African American	2054-5	2020	2020	28,203
< 1 year	0	Female	F	Hispanic	2135-2	Black or African American	2054-5	2030	2030	31,226
< 1 year	0	Female	F	Hispanic	2135-2	Native Hawaiian or Pacific Islander	2076-8	2020	2020	1,497
< 1 year	0	Female	F	Hispanic	2135-2	Native Hawaiian or Pacific Islander	2076-8	2030	2030	1,488
< 1 year	0	Female	F	Hispanic	2135-2	White	2106-3	2020	2020	449,143
< 1 year	0	Female	F	Hispanic	2135-2	White	2106-3	2030	2030	507,499
< 1 year	0	Female	F	Hispanic	2135-2	Two or More Races	MULTI	2020	2020	27,380
< 1 year	0	Female	F	Hispanic	2135-2	Two or More Races	MULTI	2030	2030	35,185
						...				
100+ years	100+	Male	M	Not Hispanic	2186-5	American Indian or Alaska Native	1002-5	2020	2020	92
100+ years	100+	Male	M	Not Hispanic	2186-5	American Indian or Alaska Native	1002-5	2030	2030	193
100+ years	100+	Male	M	Not Hispanic	2186-5	Asian	2028-9	2020	2020	683
100+ years	100+	Male	M	Not Hispanic	2186-5	Asian	2028-9	2030	2030	1,070
100+ years	100+	Male	M	Not Hispanic	2186-5	Black or African American	2054-5	2020	2020	1,953
100+ years	100+	Male	M	Not Hispanic	2186-5	Black or African American	2054-5	2030	2030	3,419
100+ years	100+	Male	M	Not Hispanic	2186-5	Native Hawaiian or Pacific Islander	2076-8	2020	2020	11
100+ years	100+	Male	M	Not Hispanic	2186-5	Native Hawaiian or Pacific Islander	2076-8	2030	2030	22
100+ years	100+	Male	M	Not Hispanic	2186-5	White	2106-3	2020	2020	15,099
100+ years	100+	Male	M	Not Hispanic	2186-5	White	2106-3	2030	2030	26,437
100+ years	100+	Male	M	Not Hispanic	2186-5	Two or More Races	MULTI	2020	2020	94
100+ years	100+	Male	M	Not Hispanic	2186-5	Two or More Races	MULTI	2030	2030	177

Exhibit A11. CDC WONDER Geographic Projections

CDC WONDER provides ten-year population projections from 2020 to 2030. The sample data below shows the projections by five-year age-band sex, and state. We mapped regions on to the states, and then used the age, sex, and region specific projections to support our modeling projections over the ten-year period.
http://wonder.cdc.gov/wonder/help/PopulationProjections.html

Age Group	Age Group Code	Gender	Gender Code	State	State Code	Year	Year Code	Projected Populations	Region
0-4 years	0-4	Female	F	Alabama	1	2020	2020	144,267	South
0-4 years	0-4	Female	F	Alabama	1	2030	2030	152,260	South
0-4 years	0-4	Female	F	Alaska	2	2020	2020	32,447	West
0-4 years	0-4	Female	F	Alaska	2	2030	2030	36,024	West
0-4 years	0-4	Female	F	Arizona	4	2020	2020	306,193	West
0-4 years	0-4	Female	F	Arizona	4	2030	2030	369,980	West
0-4 years	0-4	Female	F	Arkansas	5	2020	2020	98,414	South
0-4 years	0-4	Female	F	Arkansas	5	2030	2030	107,398	South
0-4 years	0-4	Female	F	California	6	2020	2020	1,534,935	West
				…					
85+ years	85+	Male	M	Virginia	51	2030	2030	84,778	South
85+ years	85+	Male	M	Washington	53	2020	2020	49,304	West
85+ years	85+	Male	M	Washington	53	2030	2030	74,169	West
85+ years	85+	Male	M	West Virginia	54	2020	2020	14,486	South
85+ years	85+	Male	M	West Virginia	54	2030	2030	18,683	South
85+ years	85+	Male	M	Wisconsin	55	2020	2020	46,186	Midwest
85+ years	85+	Male	M	Wisconsin	55	2030	2030	60,690	Midwest
85+ years	85+	Male	M	Wyoming	56	2020	2020	3,946	West
85+ years	85+	Male	M	Wyoming	56	2030	2030	5,867	West

Exhibit A12. T1DM Mortality Calculations

Sample Calculation for a 45 Year old Male		
**Metric**	**Value**	**Notes**
Standard Mortality Rate	0.332%	From Exhibit A9 for a 45 year old Male
T1DM Mortality Load	2.973	Load for 0-64 year olds
T1DM Mortality Rate	0.987%	Standard Mortality Rate * T1DM Mortality Load

Sample Mortality Reduction Calculation for a 45 Year old Male on CGMs and Insulin Pumps		
**Metric**	**Value**	**Notes**
Reduction Factor	50%	Reducation for CGMs and Pumps
T1DM Mortality Rate	0.987%	Calculated above
Standard Mortality	0.332%	Calculated above
T1DM mortality rate in excess of the standard mortality rate	0.655%	T1DM Mortality Rate - Standard Mortality Rate
Reduction in Mortality Rate	0.327%	Reduction Factor * T1DM mortality rate in excess of standard mortality rate
**New Mortality Rate**	**0.659%**	T1DM Mortality Rate - Reduction in Mortality Rate
